# The prognostic value of HER2 in ovarian cancer: A meta-analysis of observational studies

**DOI:** 10.1371/journal.pone.0191972

**Published:** 2018-01-30

**Authors:** Hui Luo, Xiaohui Xu, Miaomiao Ye, Bo Sheng, Xueqiong Zhu

**Affiliations:** Department of Obstetrics and Gynecology, the Second Affiliated Hospital of Wenzhou Medical University, Wenzhou, Zhejiang Province, China; University of Texas Health Science Center, UNITED STATES

## Abstract

**Background:**

The prognostic role of human epidermal growth factor receptor 2 (HER2) in ovarian cancer has been investigated in previous studies, but the results remain controversial. Here we present a meta-analysis to systematically review the association between HER2 expression and ovarian cancer prognosis.

**Method:**

Observational studies published until July 2017 were searched in Pubmed, Embase, and Cochrane library databases. Hazard ratios (HRs) for survival with 95% confidence intervals (CIs), subgroup analyses, publication bias and sensitivity analyses were implemented under a standard manner. Estimates of overall survival (OS), progress-free survival (PFS) and disease-free survival (DFS) were weighted and pooled using Der Simonian-Laird random-effect model.

**Result:**

Thirty-four studies that include 5180 ovarian cancer patients were collected for analysis. Expression of HER2 was negatively correlated with clinical prognosis of overall survival (HR = 1.57, 95% CI: 1.31 to 1.89, *P* < 0.001) and disease-free survival / progress-free survival (HR = 1.26, 95% CI = 1.06 to 1.49) in ovarian cancers. The association between HER2 expression and poor ovarian cancer prognosis in overall survival was also statistically significant in subgroups of unclassified ovarian cancer, Caucasian population and Asian population, while irrespective of detection method.

**Conclusion:**

HER2 expression was related with poor prognosis in ovarian cancer patients and can be used as a predicting cancer prognostic biomarker in ovarian cancer patients.

## Introduction

Ovarian cancer is the leading cause of gynecologic cancer death in women and impacts female life and health all over the world [1]. It is reported that ovarian cancer affects 238,719 women and causes over 150,000 deaths annually owing to that patients are diagnosed in late stages of the disease [2, 3]. Although radical surgical tumor debulking and platinum plus paclitaxel-based chemotherapy are currently established therapy of ovarian cancer patient, the prognosis of 5-year survival rate is still around 40% [4]. Hence, it is of great clinical value to identify applicable prognosis biomarkers to predict the outcomes of ovarian cancer patients.

Human epidermal growth factor receptor 2 (HER2), located on chromosome 17q12-21 [5], is a tyrosine kinase receptor in the epidermal growth factor (EGF) family and play a pivotal role in cell proliferation and tumor cell metastasis [6]. HER2 overexpression has been detected in various cancer types, including 30% of breast cancers [7], 35%-45% of pancreatic carcinomas [8], which seemed to be a poor predictor for cancer. Until now, the association between HER2 expression and ovarian cancer has been widely studied, but the results are still controversial [6, 9–41]. Most recent reports demonstrated that the expression of HER2 was a predictor of poor prognosis for ovarian cancer [12, 16, 20, 26, 34–35, 37–38, 41], while others showed that the HER2 expression had no influence on the survival in ovarian cancer patients [6, 11, 13–15, 17–19, 21–25, 27–33, 36, 39–40]. All studies assessed HER2 protein expression by immunohistochemistry or HER2 gene amplification. Therefore, to clarify a better understanding of the relationship between HER2 expression and ovarian cancer, we performed a meta-analysis combining 34 studies (5180 patients) as well as subgroups analysis, aiming to gain insights into the clinical implications.

## Materials and methods

### Search strategy

Pubmed, Embase and Cochrane library were comprehensively searched for relevant studies published from 1980 to July 2017 with the following keywords: “ovarian cancer”, “ovarian tumor”, “ovarian neoplasm”, “ovarian carcinoma” or “ovarian malignance” and “HER2”, “HER-2”, “HER 2”, “human epidermal growth factor receptor 2”, “erbB-2” or “neu” and “prognosis”, “survival” and “outcome”. No time and language restrictions were imposed. Additionally, the relevant literatures including all of the identified studies, reviews and editorials were also reviewed. All candidate studies were carried out by two independent reviewers (Luo H and Xu XH) and discrepancies were resolved by consensus.

#### Criteria for inclusion and exclusion

Studies that fulfilled the following criteria were considered eligible and selected into this article: (1) the publication explored the relation between HER2 expression and ovarian cancer prognosis, such as overall survival (OS), progress-free survival (PFS), disease-free survival (DFS) and recurrence-free survival (RFS), (2) sufficient data were either reported directly or there was sufficient data to calculate HR with 95% confidence interval (CI). (3) studies were written in English. (4) exclusion of reviews, letters to the editor, case reports and conference papers without original data. When duplicate or overlapped studies were retrieved, we included the most informative and recent articles.

### Data extraction

Two independent investigators reviewed the publications and extracted the data by aid of predefined standardized extraction forms: the first author’s name, year of publication, country of origin, histological type and stage, number of patients, detection method, age, number of HER2 expression patients and controls, follow-up time, outcome endpoint, univariate or multivariate hazard ratio (HR) and the 95% confidence interval (95% CI) for HER2 positive-expression versus HER2 negative-expression. If univariate and multivariate HR and 95%CI were both reported, multivariate results were selected in an individual study. If the article had Kaplan-Meier curves, we used Engauge Digitizer 4.1 to digitize and extract survival information from the Kaplan-Meier curves. Discrepancies were resolved by a joint consensus and discussion.

### Quality assessment

Owing to the included studies were observational studies, a Newcastle-Ottawa Scale (NOS) was used to evaluate the quality. It was used to appraise the methodological quality, which has an eight-item instrument to judge on three broad perspectives: the selection of studies; study comparability; and the ascertainment of the outcome of interest. Using the awarding of points or “stars”, we considered studies awarded with 6 or higher were classified as high-quality studies.

### Statistical analysis

MetaHR (pooled HR in the survival analysis) and 95%CI were applied to assess the association between HER2 expression and outcomes of ovarian cancer patients. Outcome endpoints were divided into two groups, OS and DFS/PFS, based on the data acquired in the current study and previous report. Statistical heterogeneity was assessed by H and I-square statistics [42], random-effects model [43–44] was used in the paper. Subgroup analysis and sensitivity analysis were performed to explore the source of heterogeneity. Publication bias was evaluated by a funnel plot with Begg’s test, if a *P* < 0.05, publication bias was probably existed. Statistical analyses were conducted Stata version 12.0 (StataCrop LP, Texas). All the statistical tests were two-sided, *P* < 0.05 was considered statistically significant.

## Results

### Eligible studies

**Fig 1:**
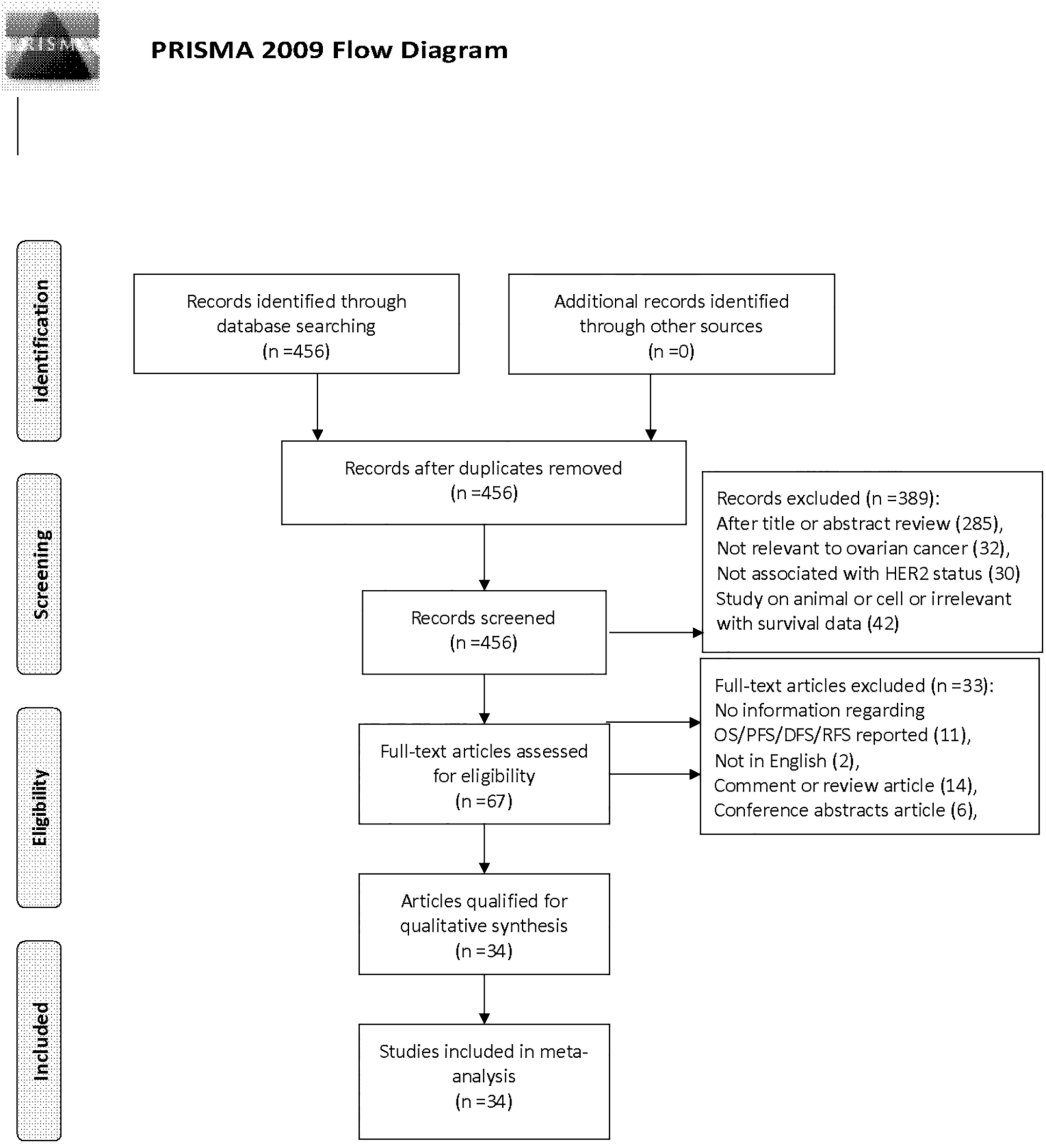
PRISMA flow chart of literature search and study selection.

A total of 456 records were retrieved from three databases by the initial search. Then 389 articles were excluded because of obvious lack of relevance. After carefully reviewing the full texts based on the inclusive criteria, 33 articles were excluded (11 had no information regarding OS/DFS/PFS, 2 studies were not written in English, 14 articles were review or comment, 6 were conference articles). Finally, 34 observational studies were selected for the present meta-analysis. A flow chart showing the study selection was presented in Fig 1.

### Demographic characteristics of included studies

The main characteristics of the 34 studies were presented in Table 1. These studies were published between 1990 and 2017. These studies were conducted in 19 countries (6 cohorts were Asian populations, 26 cohorts were Caucasian populations and 2 cohorts were mix populations). A total of 5180 patients were included with a range from 40 to 783. 27 investigations detected the HER2 status by immunohistochemistry (IHC), 3 studies used fluorescence in situ hybridization (FISH), 1 paper used chromogenic in situ hybridization (CISH), 1 research used enzyme-linked immunosorbent assay (ELISA), 1 trail used polymerase chain reaction (PCR) and the remaining 1 research used southern blot. A total of 34 studies described the association of overall survival (OS) and HER2 expression, while 14 trials involved disease-free survival (DFS) / progress-free survival (PFS). The quality of the included studies, as assessed by the Newcastle-Ottawa Scale (NOS), ranged from six to nine scores, revealing a high quality across all studies. Detailed features were recorded in Table 1.

**Table 1:** Characteristics of the included studies.

Study & year	Country	Ethnic	Histological type	Stage	Sample size	Detection method	Age (min-max)	HER2 (positive/all)	Follow up (months)	Out-comes	HR (95%CI)	Method for data collection	NOS score
Shang 2017 [9]	China	Asian	Unclassified	NA	136	IHC	54(21–83) median	41/136	48 (all)	OS	1.81 (1.16–2.83)	Directly	7
Wang 2016 [10]	China	Asian	Unclassified	I-IV	111	IHC	51.3(24–78) (mean)	35/111	NA	OS	1.92 (1.12–3.26)	Directly	7
Shandiz 2016 [11]	Iran	Asian	Unclassified	I-IV	47	IHC	51.6(19–71) (mean)	12/47	27.7(6–60) (median)	OS	0.82 (0.66–1.02)	Indirectly	7
Shandiz 2016 [11]	Iran	Asian	Unclassified	I-IV	47	IHC	51.6(19–71) (mean)	12/47	27.7(6–60) (median)	DFS	0.53 (0.04–7.57)	Indirectly	7
Zhang 2015 [12]	China	Asian	Unclassified	I-IV	161	IHC	NA	NA	60 (all)	OS	3.46 (1.84–6.52)	Directly	8
Despierre 2015 [13]	Belgium	Caucasian	Unclassified	I-IV	106	FISH	59(31–85) (median)	53/106	NA	OS	0.97 (0.49–1.89)	Directly	8
Despierre 2015 [13]	Belgium	Caucasian	Unclassified	I-IV	106	FISH	59(31–85) (median)	53/106	NA	PFS	1.51 (0.87–2.63)	Directly	8
Corkery 2015 [14]	Canada	Caucasian	Serous	NA	103	IHC	NA	NA	NA	OS	4.41 (1.95–9.95)	Indirectly	6
Corkery 2015 [14]	Canada	Caucasian	Serous	NA	103	IHC	NA	NA	NA	DFS	1.54 (0.91–2.6)	Indirectly	6
Demir 2014 [16]	Sweden	Caucasian	Unclassified	I-IV	82	IHC	54(24–80) (median)	15/82	NA	OS	4.9 (2–12.04)	Indirectly	7
Cai 2015 [6]	China	Asian	Unclassified	I-IV	95	IHC	NA	32/95	NA	OS	1.34 (0.77–2.32)	Indirectly	8
Matsuo 2014 [15]	USA	Mix	Serous	I-IV	120	IHC	62.6±10.6 (mean)	32/120	NA	OS	1.19 (0.67–2.11)	Directly	6
Matsuo 2014 [15]	USA	Mix	Serous	I-IV	120	IHC	62.6±10.6 (mean)	32/120	NA	PFS	1.04 (0.63–1.71)	Directly	6
De Toledo 2013 [17]	Brazil	Caucasian	Unclassified	I-IV	152	IHC	55.2±12.3 (mean)	19/152	43.6 (mean)	OS	1.46 (0.42–5.04)	Directly	7
De Toledo 2013 [17]	Brazil	Caucasian	Unclassified	I-IV	152	IHC	55.2±12.3 (mean)	19/152	43.6 (mean)	DFS	1.57 (0.39–6.22)	Directly	7
Chay 2013 [18]	Singapore	Mix	Serous	I-IV	113	IHC	48.3(15.8–89) (median)	31/113	2.8(0–19.99) (median)	OS	0.56 (0.21–1.52)	Directly	8
Chay 2013 [18]	Singapore	Mix	Serous	I-IV	113	IHC	48.3(15.8–89) (median)	31/113	2.8(0–19.99) (median)	PFS	0.5 (0.2–1.22)	Directly	8
Steffensen 2011 [19]	Denmark	Caucasian	Unclassified	I-IV	139	Elisa	64(32–84) (median)	NA	39.6 (median)	OS	1.11 (0.68–1.79)	Indirectly	7
Steffensen 2011 [19]	Denmark	Caucasian	Unclassified	I-IV	139	Elisa	64(32–84) (median)	NA	39.6 (median)	PFS	1.23 (0.64–2.39)	Indirectly	7
Liu 2010 [20]	China	Asian	Unclassified	I-IV	116	IHC	49(30–76) (median)	26/116	43(5–93) (median)	OS	2.83 (1.39–5.79)	Indirectly	9
Liu 2010 [20]	China	Asian	Unclassified	I-IV	116	IHC	49(30–76) (median)	26/116	43(5–93) (median)	PFS	1.92 (1–3.69)	Indirectly	9
Pfisterer 2009 [21]	Germany	Caucasian	Unclassified	IIB-IV	359	IHC	≥18	22/359	57.5(46–64.3) (median)	OS	0.71 (0.42–1.18)	Directly	8
Garcia-Velasco 2008 [23]	Spain	Caucasian	Unclassified	NA	72	IHC	57(28–82) (median)	4/72	33 (median)	OS	2.28 (0.12–4.2)	Directly	7
Garcia-Velasco 2009 [23]	Spain	Caucasian	Unclassified	NA	72	IHC	57(28–82) (median)	4/72	33 (median)	PFS	2.82 (0.38–20.9)	Directly	7
Graeff 2008 [24]	Netherland	Caucasian	Unclassified	I-IV	230	IHC	57.8(22–90) (median)	12/230	NA	OS	1.02 (0.48–2.2)	Directly	6
Graeff 2008 [24]	Netherland	Caucasian	Unclassified	I-IV	230	IHC	57.8(22–90) (median)	12/230	NA	PFS	0.98 (0.46–2.1)	Directly	6
Tomsova 2008 [22]	Czech Republic	Caucasian	Unclassified	I-IV	116	IHC	53(27–82) (median)	10/116	39(1–120) (median)	OS	1.9 (0.79–4.58)	Indirectly	7
Tuefferd 2007 [25]	France	Caucasian	Unclassified	I-IV	320	IHC	58(25–77) median	41/320	24.9 (median)	OS	0.95 (0.51–1.74)	Directly	7
Tuefferd 2007 [25]	France	Caucasian	Unclassified	I-IV	320	IHC	58(25–77) median	41/320	24.9 (median)	PFS	0.81 (0.54–1.19)	Directly	7
Steffensen 2007 [26]	Denmark	Caucasian	Unclassified	II-IV	160	IHC	54.5(29–70) median	57/160	120 (all)	OS	1.5 (1.02–2.2)	Directly	8
Pils 2007 [27]	Austria	Caucasian	Unclassified	I-IV	128	IHC	59.2 (mean)	35/128	43.7(0.4–168.7) (median)	OS	1.92 (0.94–3.94)	Indirectly	7
Malamou-Mitsi 2007[28]	Greece	Caucasian	Unclassified	I-III	95	IHC	NA	17/95	66(0.4–89.3) (median)	OS	1.85 (0.93–4.12)	Indirectly	7
Malamou-Mitsi 2007[28]	Greece	Caucasian	Unclassified	I-III	95	IHC	NA	17/95	66(0.4–89.3) (median)	PFS	1.44 (0.79–2.63)	Indirectly	7
Brozek 2006 [31]	Gdansk	Caucasian	Unclassified	I-IV	53	FISH	NA	10/53	NA	OS	2.44 (0.79–7.52)	Indirectly	6
Surowiak 2006 [29]	Germany	Caucasian	Unclassified	I-III	43	IHC	51 (mean)	21/43	0–52	OS	0.85 (0.17–4.33)	Indirectly	7
Castellvi 2006 [30]	Spain	Caucasian	Unclassified	I-IV	75	IHC	55(20–87) (mean)	23/75	31(24–80) (mean)	OS	1.12 (0.49–2.54)	Indirectly	7
Verri 2005 [32]	Italy	Caucasian	Unclassified	I-IV	194	IHC	57(25–90) median	53/194	45(1–161) (median)	OS	1.36 (0.76–2.42)	Directly	8
Verri 2005 [32]	Italy	Caucasian	Unclassified	I-IV	194	IHC	57(25–90) median	53/194	45(1–161) (median)	PFS	1.61 (0.94–2.73)	Directly	8
Lassus 2004 [34]	Sweden	Caucasian	Serous	I-IV	401	CISH	NA	66/401	NA	OS	2.14 (1.34–3.42)	Directly	7
Nielsen 2003 [33]	Denmark	Caucasian	Unclassified	I-IV	783	IHC	58(13–91) (median)	272/783	NA	OS	0.95 (0.66–1.36)	Directly	7
Camilleri-Broet 2004 [35]	France	Caucasian	Unclassified	IIIA-IV	95	IHC	59(23–74) median	15/95	68 (median)	OS	2.12 (1.13–3.98)	Directly	7
Camilleri-Broet 2004 [35]	France	Caucasian	Unclassified	IIIA-IV	95	IHC	59(23–74) median	15/95	68 (median)	PFS	1.99 (1.12–3.54)	Directly	7
Skirnisdottir 2001[36]	Sweden	Caucasian	Unclassified	IA-IIC	106	IHC	60(26–82) mean	20/106	87(57–125) mean	OS	2.28 (0.67–7.82)	Indirectly	8
Davidson 2000 [37]	Norway	Caucasian	Unclassified	NA	75	IHC	56.9(30–84) mean	35/75	70(8–224) mean	OS	1.92 (1.1–3.37)	Indirectly	8
Wang 1999 [38]	USA	Caucasian	Unclassified	NA	40	FISH	61(35–83) median	10/40	1–56 all	OS	4 (1.2–13.9)	Indirectly	8
Medl 1995 [39]	Austria	Caucasian	Unclassified	I-IV	196	PCR	59.6(15–88) median	79/196	59 mean	OS	1.08 (0.73–1.6)	Indirectly	8
Fajac 1995[40]	France	Caucasian	Unclassified	I-IV	65	South blot	52 mean	9/65	71 (10–43) median	OS	1.8 (0.75–4.33)	Indirectly	7
Berchuck 1990 [41]	USA	Caucasian	Unclassified	NA	73	IHC	63.5 (median)	23/73	1–100 (all)	OS	4.39 (2.13–9.06)	Inirectly	8

Abbreviations: HR: hazard ratio, CI: confidence interval, IHC: immunohistochemistry, FISH: fluorescence in situ hybridization. ELISA: enzyme-linked immunosorbent assay, CISH: chromogenic in situ hybridization, PCR: polymerase chain reaction, NA: not available, OS: overall survival, DFS/PFS: disease-free survival/ progress-free survival, Serous: serous ovarian cancer, Unclassified: serous, mucinous, clear cell, endometrioid, transitional cell, undifferentiated, differentiated, and others.

### Association of HER2 expression with overall survival and its subgroup analysis

**Fig 2:**
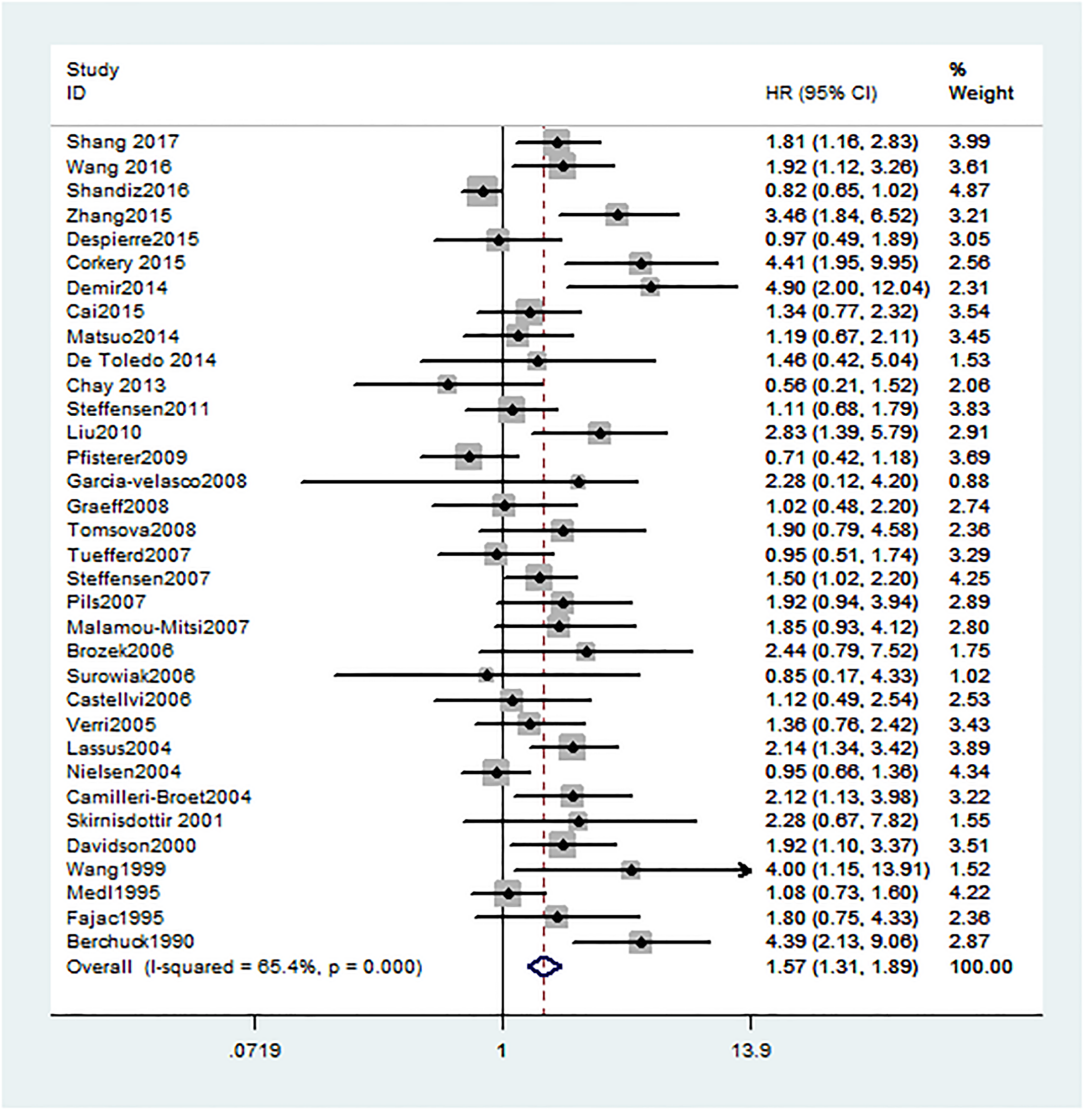
Forest plots of HR and 95%CI for overall survival in ovarian cancer according to presence of HER2. Random-effects model was used.

All 34 studies investigating OS were showed that HER2 positive expression in ovarian cancer patients was significantly associated with worse OS (HR = 1.57, 95% CI: 1.31 to 1.89, H^2^ = 1.7). As severe heterogeneity was observed (I^2^ = 65.4%, 95%CI: 50% to 76%), a random-effects model was determined for the pooled HR and 95% CI and subgroup meta-analysis was conducted to investigate the possible source of the heterogeneity among studies (Fig 2).

**Fig 3:**
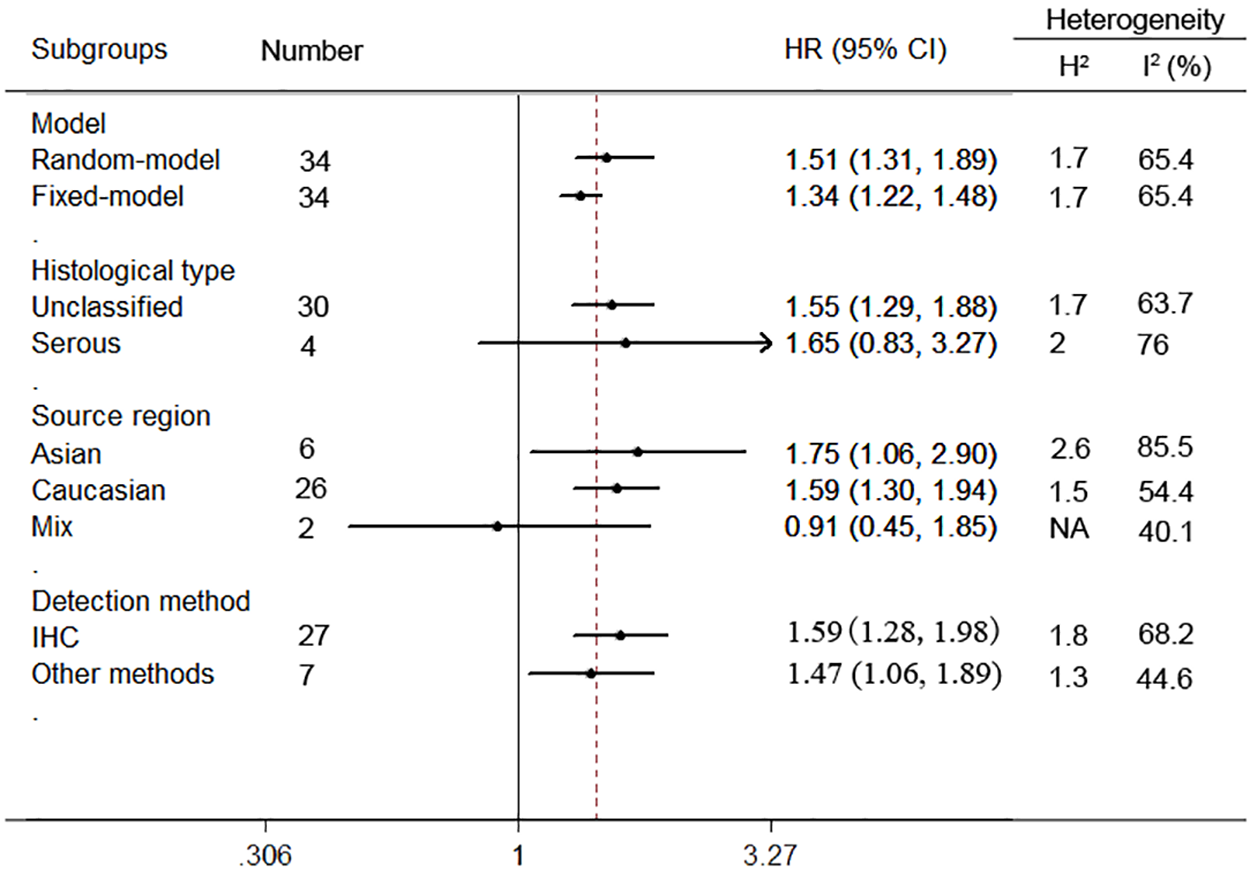
Subgroup analyses of the relationship between HER2 expression and overall survival of ovarian cancer.

In the stratified analysis by histological type, HER2 expression was associated with worse OS of unclassified ovarian cancer (n = 30, HR = 1.55, 95% CI = 1.29 to 1.88, H^2^ = 1.7; I^2^ = 63.7%, 95%CI = 46% to 75%), while HER2 expression implied no significant association in serous ovarian cancer (n = 4, HR = 1.65, 95% CI = 0.83 to 3.27, H^2^ = 2; I^2^ = 76%, 95%CI = 34% to 91%) (Fig 3).

When sub-grouped by ethnicity, a worse overall survival was strong linked to HER2 positivity in Asian populations (n = 6, HR = 1.75, 95% CI = 1.06 to 2.9, H^2^ = 2.6; I^2^ = 85.5%, 95%CI = 70% to 93%) as well as Caucasian populations (n = 26, HR = 1.59, 95% CI = 1.3 to 1.94, H^2^ = 1.5; I^2^ = 54.4%, 95%CI = 29% to 71%). Nevertheless, HER2 positivity was irrelevant to OS of ovarian cancer in Mix populations (n = 2, HR = 0.91, 95% CI = 0.45 to 1.85, I^2^ = 40.1%).

With regard to different detection methods of HER2 in ovarian cancer, positive HER2 expression status was a worse prognostic marker of overall survival in immunohistochemistry (IHC) group (n = 27, HR = 1.59, 95% CI = 1.28 to 1.98, H^2^ = 1.8; I^2^ = 68.2%, 95% CI = 54% to 79%). Similarly, HER2 expression was also associated with OS by using other detection methods (n = 7, HR = 1.47, 95% CI = 1.06 to 1.89, H^2^ = 1.3; I^2^ = 44.6%, 95% CI = 0% to 77%).

### Association of HER2 expression with disease-free survival / progress-free survival and its subgroup analysis

**Fig 4:**
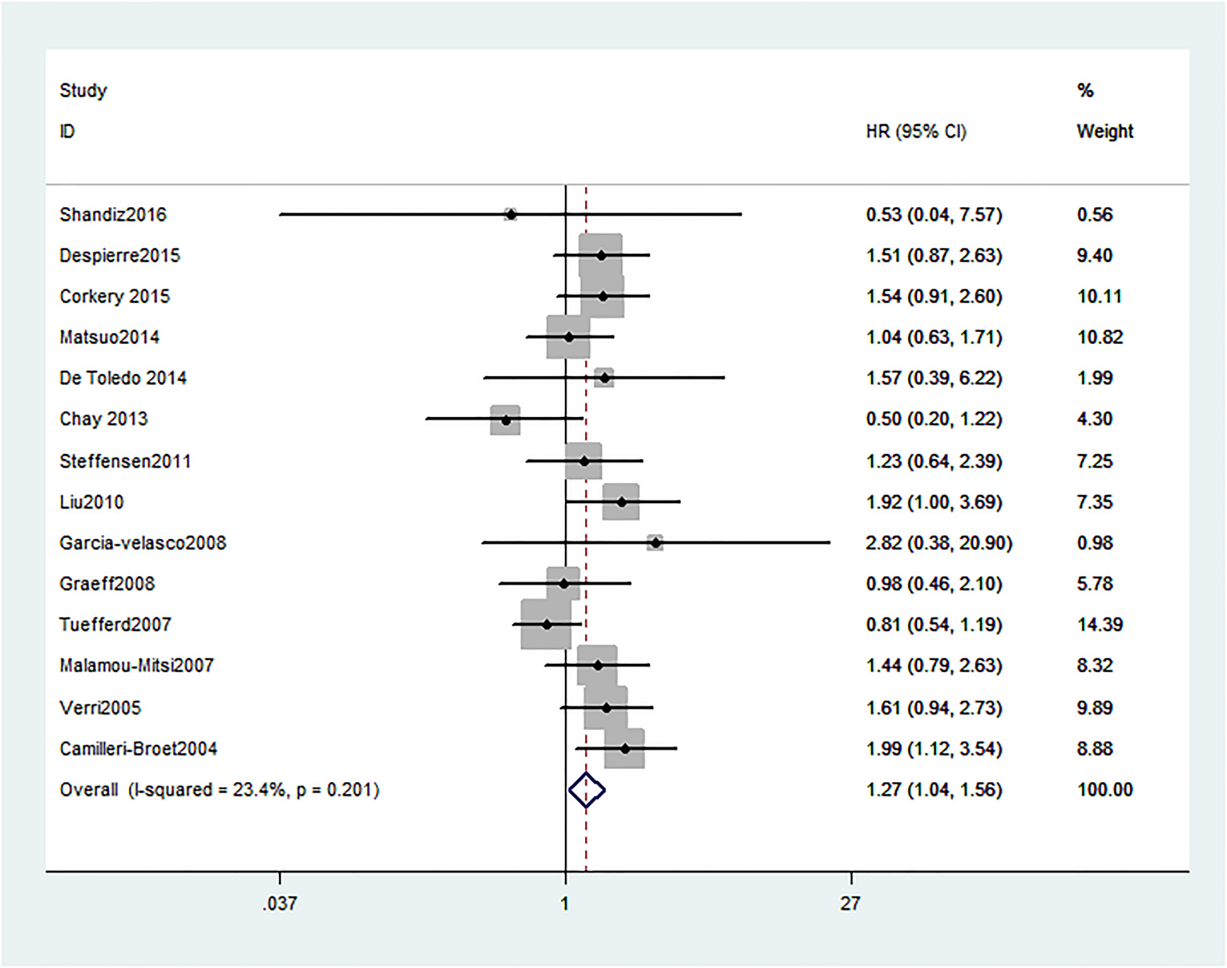
Forest plots of HR and 95%CI for disease-free survival / progress-free survival in ovarian cancer according to presence of HER2. Random-effects model was used.

Pooled HRs and 95% CI for disease-free survival (DFS) / progress-free survival (PFS) were conducted in 14 studies, the pooling analysis showed an increased risk of disease progression in patients with HER2 positive group (HR = 1.27, 95% CI = 1.04 to 1.56), along with a moderate heterogeneity of the data (I^2^ = 23.4%, 95% CI = 0% to 59%) (Fig 4).

**Fig 5:**
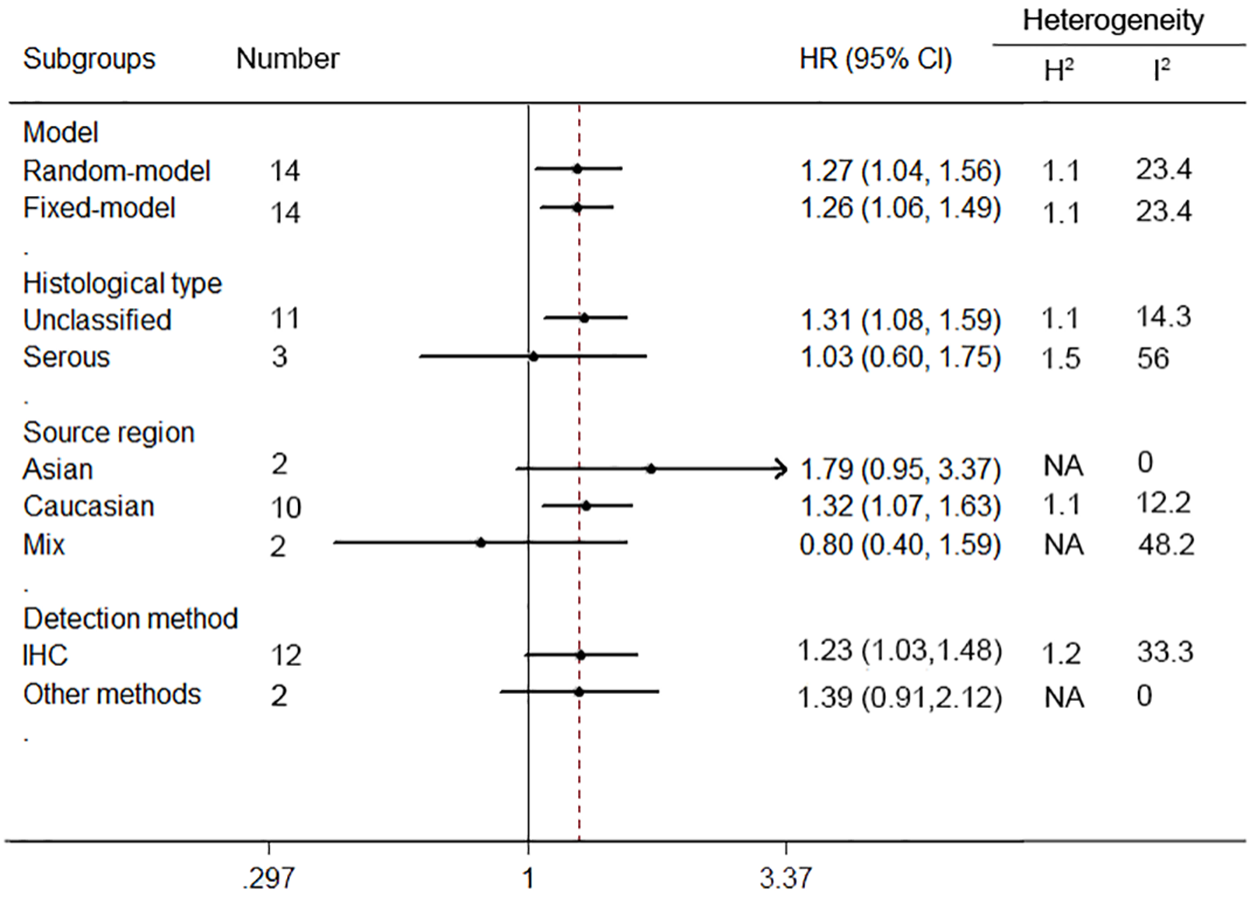
Subgroup analyses of the relationship between HER2 expression and disease-free survival / progress-free survival of ovarian cancer.

When considering differences in histological types of cancers, high levels of HER2 were significantly associated with a poorer DFS/PFS of unclassified ovarian cancer patients (n = 11, HR = 1.34, 95% CI = 1.08 to 1.67, H^2^ = 1.1; I^2^ = 14.3%, 95% CI = 0% to 55%), but not in serous ovarian cancer patients (n = 3, HR = 1.03, 95% CI = 0.6 to 1.75, H^2^ = 1.5; I^2^ = 56%, 95% CI = 0% to 87%) (Fig 5).

Subgroup analyses by ethnicity revealed that HER2 was an unfavorable predictor of DFS/PFS in Caucasian populations (n = 10, HR = 1.32, 95% CI = 1.07 to 1.63, H^2^ = 1.1; I^2^ = 12.2%, 95% CI = 0% to 53%). However, not significant association between positive HER2 expression and poor DFS/PFS was found in Asian populations (n = 2, HR = 1.79, 95% CI = 0.95 to 3.37, I^2^ = 0%) or Mix populations (n = 2, HR = 0.8, 95% CI = 0.4 to 1.59, I^2^ = 48.2%).

Among the subgroups determined by detection approaches, HER2 over-expression in IHC detection group was related to a significantly worse DFS/PFS (n = 12, HR = 1.23, 95% CI = 1.03 to 1.48, H^2^ = 1.2, I^2^ = 33.3%, 95% CI = 0% to 66%), whereas, there was no significant association between HER2 expression and DFS/PFS among patients in the other detection methods groups (n = 2, HR = 1.39, 95% CI = 0.91 to 2.12, I^2^ = 0%).

### Publication bias

**Fig 6:**
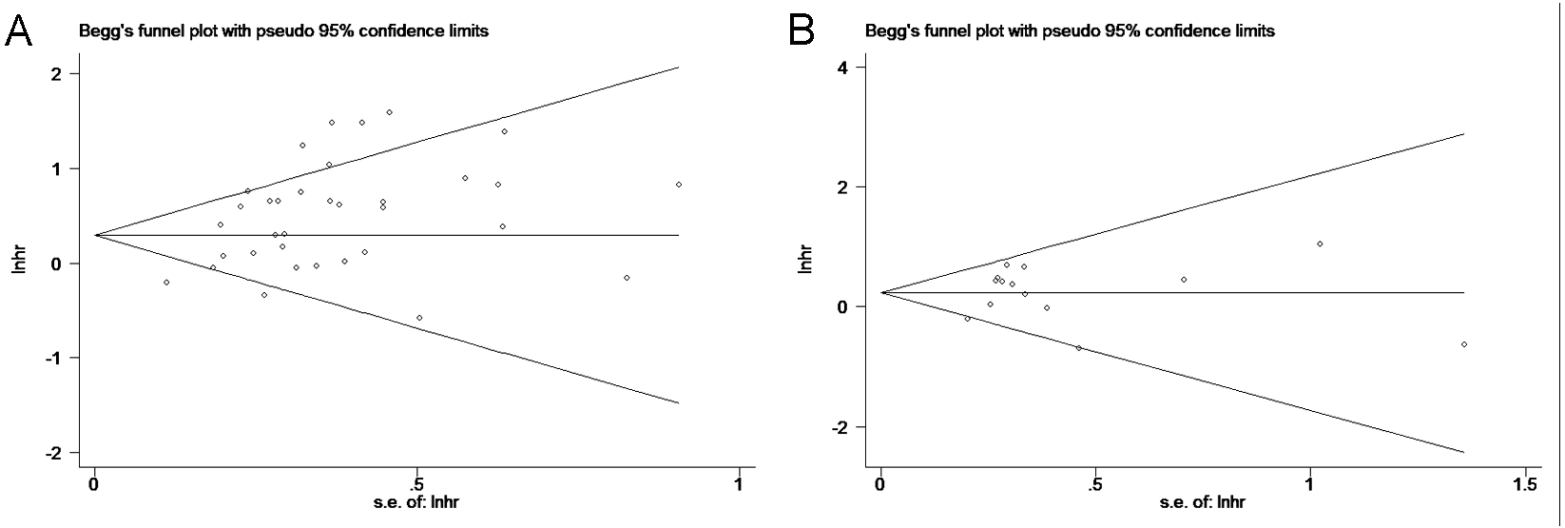
**6A.** Begg’s publication bias plot of the studies assessing HER2 expression and overall survival in ovarian cancer. **6B.** Begg’s publication bias plot of the studies assessing HER2 expression and disease-free survival / progress-free survival in ovarian cancer. Visual inspection of the funnel plot did not identify substantial asymmetry.

Begg’s test was used to investigate publication bias. No evidence of publication bias was observed for OS (*P* = 0.192) or DFS/PFS (*P* = 0.827) analyses (Fig 6A and 6B).

### Sensitivity analysis

**Fig 7:**
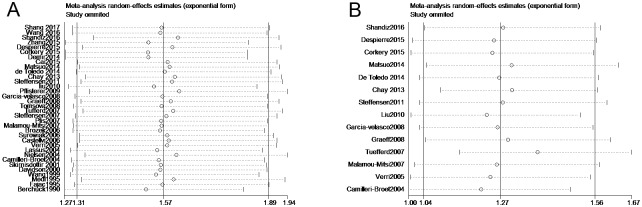
**7A.** Sensitivity analysis of 34 studies included in this meta-analysis for overall survival. **7B.** Sensitivity analysis of 14 studies included in this meta-analysis for disease-free survival / progress-free survival. Leave-one-out method was used to confirm the stability of the results.

A leave-one-out sensitivity analysis by removing sequential study per time was adopted to assess the influence of each study on the pooled HR (Fig 7A and 7B). The result was not obviously changed when any single study was elided.

## Discussion

To our knowledge, this is the most comprehensive meta-analysis of the current literature on HER2, although our result is consistent with the only previous study to explore the prognostic role of HER2 in ovarian cancer in 2013 [45]. Notably, our research included almost four times more patients than the previously reported one, and the studies employed more subgroups and patients with longer follow-ups. Therefore, our meta-analysis was able to show a more reliable result.

In the current meta-analysis, we systematically evaluated survival data from 34 studies, which including 5180 patients. We demonstrated that the expression of HER2 was an indicator of a poor prognosis of ovarian cancer, with consistent results of OS and DFS/PFS. HER2 expression was low in normal ovarian epithelium while expressed highly in a variable percentage of epithelial ovarian cancer (11%-66%) [39, 46]. Either gene amplification or overexpression may lead to the dysregulation of HER2 signaling in ovarian cancer, then result in faster cell growth, DNA damage and increasing tumor progression [47]. Such effects may partially explain the negative relationship between HER2 expression and survival rate of ovarian cancer patients.

Ovarian cancers consist of many histological subtypes, including those of serous, mucinous, endometrioid and clear cell cancer [9]. The expression of tumor biomarkers was different according to clinicopathological features, including histological types. Shang et al. [9] found HER2 positivity was much higher in serous (29%) and mucinous carcinoma (38%) than that in endometrioid (20%) and clear cell carcinoma (23.1%). In the present study, we found that with respect to histological types, increased levels of HER2 had a negative influence on OS and DFS/PFS in the unclassified ovarian cancers. Corkery et al. [14] and Lassus et al. [34] presented an association of HER2 with poor survival in serous ovarian carcinoma, but the pooled four articles [14–15, 18, 34] showed that HER2 expression was related to neither OS nor DFS/PFS in serous type of ovarian cancer. Therefore, we suggest that the expression of HER2 may be a prognostic biomarker in non-serous ovarian cancer rather than serous ovarian cancer.

Regarding the ethnicity/race, HER2 expression was correlated with poorer OS of ovarian cancer patients in Asian group and Caucasian group but not in mix populations. Nevertheless, HER2 expression implied a worse PFS/DFS trend in Caucasian populations and showed no significant association in Asian populations or mix populations. It seemed that certain genes exerted different effects on cancer risk and prognosis across ethnic group. For instance, patient with high expression of HER2 lle655Val polymorphism have a negative prognosis among Caucasian subgroup, while no significant associations were observed in the Asian and African groups [48]. These maybe caused by genetic background, life style and environmental effect differed from ethnic regions.

Subgroup analysis showed that expression of HER2 had a negative influence on clinical outcome in the immunohistochemical technology group, with consistent results of OS and DFS/PFS. Nevertheless, in other detection method, HER2 expression implied poor OS outcome while showed no association with DFS/PFS/RFS. However, it was still difficult to draw a conclusion because the result was based on small numbers and required confirmation in large studies. These two studies detected HER2 using fluorescence in situ hybridization (FISH) and enzyme-linked immunosorbent assay (ELISA). FISH was a molecular based technique that detected HER2 gene amplification, but HER2 protein overexpression was attributable to gene amplification, what’s more, copy number intensity of signal was reflective of the quality of HER2 protein, on the other hand, FISH was a valid and supplement method to reflect HER2 overexpression to recommend trastuzumab therapy [49].

Immunohistochemical staining was widely used to detect the distribution and localization of biomarkers and protein expression status in the biological tissue and contributes to decisions on prognosis.

There are several important implications for the clinical management of ovarian cancer. First, it shows that HER2 expression is associated with worse outcome of ovarian cancer, implicating HER2 maybe a potential prognostic indicator for ovarian cancer patients. Second, it identifies a subgroup of ovarian cancer with histological type, source region and detection technology to analyze the heterogeneity. Finally, publication bias tests and plots are only relevant if studies are more than 10 otherwise underpowered to detect much and tend to lead to conclusions [50], in our study, there were 34 studies and it was considerable strength, which indicated the statistical results of the analyses were robust.

Some limitations in this meta-analysis have to be mentioned. First, it based on population-level data rather than individual patient-level data. Second, some of the HRs and 95% CIs were extracted indirectly from growth curve or formula computing, which could result in bias of outcome in certain extent. Third, due lack of detailed data, we only performed the sub-group analysis between HER2 and ovarian cancer with OS or PFS/DFS. Therefore, further investigations are needed to address these shortcomings.

## Conclusion

In summary, our study suggests that HER2 may be a potential marker to predict the poor prognosis of ovarian cancer patients, especially for patients with unclassified ovarian cancer and Caucasian region. Additionally, immunohistochemistry is an effective method for predicting ovarian cancer clinical outcomes when evaluate HER2 expression.

## Supporting information

S1 Table PRISMA checklist. (DOC)
