# Expression of Concern: The prognostic value of HER2 in ovarian cancer: A meta-analysis of observational studies

**DOI:** 10.1371/journal.pone.0279960

**Published:** 2022-12-30

**Authors:** The PLOS ONE Editors

After this article was published, similarities were noted between this article [1] and submissions by other research groups which call into question the validity and provenance of the reported results.

In response to queries about these concerns, the corresponding author provided the underlying data in S1 File. They also stated that they did not receive any third-party support in conducting this research, analysing the data, or in preparing the manuscript for submission.

The authors commented on aspects of how data were collected and analyzed for this study, but overall their responses did not fully resolve the concerns.

The *PLOS ONE* Editors issue this Expression of Concern to notify readers of the unresolved concerns discussed above, and to provide the data received from the authors.

## Supporting information

S1 File The underlying data used for the analysis in this article. Pubmed, Embase and Cochrane library databases were searched up until 2017, the results are shown in the list in folder “Search strategy”. These results were imported into Endnote resulting in 34 articles being included in this study (folder “Full texts extraction”). Data were extracted into Excel (file “HER2 New”), including hazard ratios (HRs) for survival with 95% confidence intervals (CIs). Subgroup analyses (file “HER2 New”), publication bias and sensitivity analyses (folder “Figures”) were carried out. Estimates of overall survival (OS), progress-free survival (PFS) and disease-free survival (DFS) were weighted and pooled using Der Simonian-Laird random-effect model (file “HER2 New”). Stata was used to draw figures (folder “Figures”).
(ZIP)
