# Supplementary material for: Expression of Concern: The prognostic value of HER2 in ovarian cancer: A meta-analysis of observational studies
Source: PLoS One. 2022 Dec 30;17(12):e0279960. doi: 10.1371/journal.pone.0279960 (PMC9803123; doi:10.1371/journal.pone.0279960)
Supplement: S1 File — Pubmed, Embase and Cochrane library databases were searched up until 2017, the results are shown in the list in folder “Search strategy”. These results were imported into Endnote resulting in 34 articles being included in this study (folder “Full texts extraction”). Data were extracted into Excel (file “HER2 New”), including hazard ratios (HRs) for survival with 95% confidence intervals (CIs). Subgroup analyses (file “HER2 New”), publication bias and sensitivity analyses (folder “Figures”) were carried out. Estimates of overall survival (OS), progress-free survival (PFS) and disease-free survival (DFS) were weighted and pooled using Der Simonian-Laird random-effect model (file “HER2 New”). Stata was used to draw figures (folder “Figures”). (ZIP) [file pone.0279960.s001.zip › HER2 data (1)/HER2 data/~WRL1917.tmp]

**Introduction**

Ovarian cancer is the leading cause of gynecologic cancer death in women and impacts female life and health all over the word[1]. It is reported that ovarian cancer affects 238,719 women and causes over 150,000 deaths annually owing to that patients are diagnosed in late stages of the disease[2, 3]. Although radical surgical tumor debulking and platinum plus paclitaxel-based chemotherapy are currently established therapy of ovarian cancer patient, the prognosis of 5-year survival rate is still around 40%[4]. Hence, it is of great clinical value to identify applicable prognosis biomarkers to predict the outcomes of patients.

Human epidermal growth factor receptor 2 (HER2), located on chromosome 17q12-21[5], is a tyrosine kinase receptor in the epidermal growth factor (EGFR) family and play a pivotal role in cell proliferation and tumor cell metastasis[6].

**Materials and methods**

**Search strategy**

Pubmed, Embase and Cochrane library were comprehensively searched for relevant studies published until July 2017 with the following keywords: “ovarian cancer”, “ovarian tumor”, “ovarian neoplasm” or “ovarian cancer” and “HER2”, “HER-2”, “human epidermal growth factor receptor 2” or “erbB-2” and “prognosis”, “survival” and “outcome”. No time and language restrictions were imposed. Additionally, the relevant literatures including all of the identified studies, reviews and editorials were also reviewed. All candidate studies were carried out by two independent reviewers (Luo H and Xu XH) and discrepancies were resolved by consensus.

**Selection criteria**

Studies that fulfilled the following criteria were considered eligible and selected into this article: (1) the publication explored the relation between HER2 expression and ovarian cancer prognosis, such as OS, PFS, DFS and RFS, (2) sufficient data were either reported directly or there was sufficient data to calculate HR with 95% confidence interval (CI). (3) studies were written in English. (4) exclusion of reviews, letters to the editor, case reports and conference papers without original data. When duplicate or overlapped studies were retrieved, we included the most informative and recent article.

**Data extraction**

Two independent investigators reviewed the publications and extracted the data by aid of predefined standardized extraction forms: the first author’s name, year of publication, country of origin, histological type, number of patients, age, detection method, number of HER2 over-expression patients and controls, follow-up time, outcome endpoint, univariate or multivariate hazard ratio (HR) and the 95% confidence interval (95% CI) for HER2 positive-expression versus HER2 negative-expression. If univariate and multivariate HR and 95%CI were both reported, multivariate results were selected in an individual study. If HR was not displayed directly, we used Engauge Digitizer 4.1 to digitize and extract survival information from the Kaplan-Meier curves. Discrepancies were resolved by a joint consensus and discussion.

**Quality assessment**

Owing to the included studies were observational studies, a Newcastle-Ottawa Scale (NOS) was used to evaluated the quality, NOS scores of 6 or higher were classified as high-quality studies.

**Statistical analysis**

MetaHR and 95%CI were applied to assess the association between HER2 expression and outcomes of ovarian cancer patients. Outcome endpoints were divided into two groups, OS and DFS/RFS/PFS, based on the data acquired in the current study and previous report. Statistical heterogeneity was assessed by Cochrane’s Q and I-square statistics. If Cochrane’s Q test with *P* < 0.1 or I^2^ > 50%, it was considered a severe heterogeneity, then the random-effects model was used; otherwise, the fixed-effects model was chosen. Subgroup analysis and sensitivity analysis were performed to explore the source of heterogeneity. Publication bias was evaluated by a funnel plot with Egger’s test, if a *P* < 0.05, publication bias was probably existed. Statistical analyses were conducted STATA version 12.0 (StataCrop LP, Texas). All the statistical tests were two-sided, *P* < 0.05 was considered statistically significant.

**Results**

**Eligible studies**

A total of 456 records were retrieved from three databases by the initial search. Then 389 articles were excluded because of obvious lack of relevance. After carefully reviewing the full texts based on the inclusive criteria, 33 articles were excluded (11 had no information regarding OS/DFS/PFS/RFS, 2 studies were not written in English, 14 articles were review or comment, 6 were conference articles). Finally, 34 studies were selected for the present meta-analysis. A flow chart showing the study selection was presented in Figure 1.

**Demographic characteristics of included studies**

The main characteristics of the 34 studies were presented in Table 1. These studies were published between 1990 and 2017. Among these studies: 20 studies from Europe, 7 from Asia, 4 from North America, 2 from Oceania and 1 from South America []. A total of 5180 patients were included with a range from 40 to 783. 26 investigations detected the HER2 status by immunohistochemistry (IHC), 3 studies used fluorescence in situ hybridization (FISH), 1 paper used chromogenic in situ hybridization (CISH), 1 research used enzyme-linked immunosorbent assay (ELISA), 1 study used immunostaining, 1 trail used PCR and the remaining 1 research used southern blot. A total of 34 studies described the correlation of overall survival (OS) and HER2 expression, while 14 trials involved disease-free survival (DFS) / progress-free survival (PFS) / recurrence-free survival (RFS). The quality of the included studies, as assessed by the Newcastle-Ottawa Scale (NOS), ranged from five to eight scores, revealing a high quality across all studies. Detailed features were recorded in Table 1.

**Correlation of HER2 expression with overall survival and its subgroup analysis**

All 34 studies investigating OS were showed that HER2 positive expression in ovarian cancer patient was significantly associated with worse OS (HR=1.57, 95% CI: 1.31 to 1.89, *P* < 0.001). As moderate heterogeneity was observed (I^2^ = 65.4%), so that a random-effects model was determined for the pooled HR and 95% CI and subgroup meta-analysis was conducted to investigate the possible source of the heterogeneity among studies (Figure 2)

In the stratified analysis by histological type, HER2 expression was associated with worse OS of unclassified ovarian cancer (n = 30, HR = 1.55, 95% CI = 1.29 to 1.88, *P* = 0.000, I^2^ = 63.7%), while HER2 expression implied no significant association in serous ovarian cancer (n = 4, HR= 1.65, 95% CI = 0.83 to 3.27, *P* = 0.006, I^2^ = 72%).

When sub-grouped by ethnicity, a worse overall survival was strong linked to HER2 positivity in European population (n = 20, HR = 1.36, 95% CI = 1.19 to 1.57, *P* = 0.026, I^2^ = 41.8%) as well as north American group (n = 4, HR = 2.94, 95% CI = 1.36 to 6.33, *P* = 0.011, I^2^ = 73.1%). Nevertheless, HER2 positivity was irrelevant to OS of ovarian cancer in Asian population, south American group and Oceanian group.

With regard to different detection methods of HER2 in ovarian cancer, high HER2 expression status was a worse prognostic marker of overall survival in immunohistochemistry (IHC) group (n = 26, HR = 1.62, 95%CI = 1.3 to 2.02, *P* = 0.000, I^2^ = 70.2%). Similarly, HER2 expression was also associated with OS by using other detection methods (n = 8, HR = 1.36, 95%CI = 1.1 to 1.68, *P* = 0.122, I^2^ = 38.7%).

**Correlation of HER2 expression with DFS/PFS/RFS and its subgroup analysis**

HRs and 95% CI for DFS/PFS/RFS were conducted in 14 studies, the pooling analysis showed an increased risk of disease progression in patients with HER2 positive group, both in random (HR = 1.27, 95% CI = 1.04 to 1.56) and fixed model (HR = 1.26, 95% CI = 1.06 to 1.49), along with a moderate heterogeneity of the data (I^2^ = 23.4%), hence, fixed-model was used to determine the summary of outcomes (Figure ).

When considering differences in histological types of cancers, high levels of HER2 were significantly associated with a poorer DFS/PFS/RFS of unclassified ovarian cancer patients (n= 11, HR = 1.31, 95% CI = 1.08 to 1.59, *P* = 0.308, I^2^ = 14.3%), but not in serous ovarian cancer patients (n = 3, HR = 1.03, 95% CI = 0.6 to 1.75, *P* = 0.103, I^2^ = 56%) (Figure ).

Subgroup analyses by ethnicity revealed that HER2 was an unfavorable predictor of DFS/PFS/RFS in European populations (n = 8, HR = 1.26, 95%CI = 1.03 to 1.56, *P* = 0.205, I^2^ = 27.9%). However, not significant association between high HER2 expression and poor DFS/PFS/RFS was found in Asian group (n = 3, HR = 0.94, 95% CI = 0.31 to 2.83, *P* = 0.051, I^2^ = 66.4%), north American group (n = 2, HR = 1.25, 95% CI = 0.87 to 1.8, *P* = 0.221, I^2^ = 11.3%) and south American group (n = 1, HR = 1.57, 95% CI = 0.39 to 6.22).

Among the subgroups determined by detection approaches, HER2 over-expression in IHC group contributed to a significantly worse DFS/PFS/RFS prognosis (n = 11, HR = 1.25, 95% CI = 1.03 to 1.51, *P* = 0.096, I^2^ = 38%), Moreover, HER2 positive expression using other detection group likewise suggested a poor DFS/PFS/RFS outcome (n = 3, HR = 1.28, 95% CI = 0.88 to 1.85, *P* = 0.66, I^2^ = 0%).

**Publication bias**

Begg’s and Egger’s tests were used to investigate publication bias. No evidence of publication bias was observed for OS (Begg: P = 0.192, Egger: P = 0.187) or DFS/PFS/RFS (Begg: P = 0.827, Egger: P = 0.784) analyses.

**Sensitivity analysis**

Leave-one-out sensitivity analyses by removing sequential study per time was adopted to assess the influence of each study on the pooled HR (Figure ). The result was not obviously changed when any single study was elided.

[1]. Jemal A, Bray F, Center MM, Ferlay J, Ward E, Forman D. Global cancer statistics[J]. CA Cancer J Clin. 2011,61(2):69-90.

[2]. Chudecka-Glaz AM. ROMA, an algorithm for ovarian cancer[J]. Clin Chim Acta. 2015,440:143-51.

[3]. Jacobs IJ, Menon U. Progress and challenges in screening for early detection of ovarian cancer[J]. Mol Cell Proteomics. 2004,3(4):355-66.

[4]. Liu J, Matulonis UA. New strategies in ovarian cancer: translating the molecular complexity of ovarian cancer into treatment advances[J]. Clinical cancer research : an official journal of the American Association for Cancer Research. 2014,20(20):5150-6.

[5]. Schluter B, Gerhards R, Strumberg D, Voigtmann R. Combined detection of Her2/neu gene amplification and protein overexpression in effusions from patients with breast and ovarian cancer[J]. Journal of cancer research and clinical oncology. 2010,136(9):1389-400.

[6]. Cai Y, Wang J, Zhang L, Wu D, Yu D, Tian X, et al. Expressions of fatty acid synthase and HER2 are correlated with poor prognosis of ovarian cancer[J]. Medical oncology (Northwood, London, England). 2015,32(1):391.
