# Supplementary material for: Expression of Concern: The prognostic value of HER2 in ovarian cancer: A meta-analysis of observational studies
Source: PLoS One. 2022 Dec 30;17(12):e0279960. doi: 10.1371/journal.pone.0279960 (PMC9803123; doi:10.1371/journal.pone.0279960)
Supplement: S1 File — Pubmed, Embase and Cochrane library databases were searched up until 2017, the results are shown in the list in folder “Search strategy”. These results were imported into Endnote resulting in 34 articles being included in this study (folder “Full texts extraction”). Data were extracted into Excel (file “HER2 New”), including hazard ratios (HRs) for survival with 95% confidence intervals (CIs). Subgroup analyses (file “HER2 New”), publication bias and sensitivity analyses (folder “Figures”) were carried out. Estimates of overall survival (OS), progress-free survival (PFS) and disease-free survival (DFS) were weighted and pooled using Der Simonian-Laird random-effect model (file “HER2 New”). Stata was used to draw figures (folder “Figures”). (ZIP) [file pone.0279960.s001.zip › HER2 data (1)/HER2 data/Figures/Figure1.doc]

**PRISMA 2009 Flow Diagram**

**Screening**

**Included**

**Eligibility**

**Identification**

Records identified through database searching
(n =456)

Additional records identified through other sources
(n =0)

Records after duplicates removed
(n =456)

Records screened
(n =456)

Records excluded
(n =389)

Full-text articles assessed for eligibility
(n =67)

Full-text articles excluded,
(n =33)

No information regarding OS/PFS/DFS/RFS reported (11)

Not in English (2)

Comment or review article (14)

Conference abstracts article (6)

Articles qualified for qualitative synthesis
(n =34)

Studies included in meta-analysis
(n =34)
