# Supplementary material for: Expression of Concern: The prognostic value of HER2 in ovarian cancer: A meta-analysis of observational studies
Source: PLoS One. 2022 Dec 30;17(12):e0279960. doi: 10.1371/journal.pone.0279960 (PMC9803123; doi:10.1371/journal.pone.0279960)
Supplement: S1 File — Pubmed, Embase and Cochrane library databases were searched up until 2017, the results are shown in the list in folder “Search strategy”. These results were imported into Endnote resulting in 34 articles being included in this study (folder “Full texts extraction”). Data were extracted into Excel (file “HER2 New”), including hazard ratios (HRs) for survival with 95% confidence intervals (CIs). Subgroup analyses (file “HER2 New”), publication bias and sensitivity analyses (folder “Figures”) were carried out. Estimates of overall survival (OS), progress-free survival (PFS) and disease-free survival (DFS) were weighted and pooled using Der Simonian-Laird random-effect model (file “HER2 New”). Stata was used to draw figures (folder “Figures”). (ZIP) [file pone.0279960.s001.zip › HER2 data (1)/HER2 data/Figures/Table 1.doc]

**Table 1. Characteristics of the studies included in this meta-analysis.**

| **Study & year** | **Country** | **Histological**  **type** | **Stage** | **Sample size** | **Detection method** | **Age**  **(min-max)** | **HER2**  **(positive/all)** | **Follow up (months)** | **Out-**  **comes** | **HR**  **(95%CI)** | **Method for data collection** | **NOS**  **score** |
| --- | --- | --- | --- | --- | --- | --- | --- | --- | --- | --- | --- | --- |
| Shang  2017 [9] | China | Unclassified | NA | 136 | IHC | 54(21-83)  median | 41/136 | 48  (all) | OS | 1.81  (1.16-2.83) | Directly | 7 |
| Wang  2016 [10] | China | Unclassified | I-IV | 111 | IHC | 51.3(24-78)  (mean) | 35/111 | NA | OS | 1.92  (1.12-3.26) | Directly | 7 |
| Shandiz  2016 [11] | Iran | Unclassified | I-IV | 47 | IHC | 51.6(19-71)  (mean) | 12/47 | 27.7(6-60)  (median) | OS | 0.82 (0.66-1.02) | Indirectly | 7 |
| Shandiz  2016 | Iran | Unclassified | I-IV | 47 | IHC | 51.6(19-71)  (mean) | 12/47 | 27.7(6-60)  (median) | DFS | 0.53  (0.04-7.57) | Indirectly | 7 |
| Zhang  2015 | China | Unclassified | I-IV | 161 | IHC | NA | NA | 60  (all) | OS | 3.46  (1.84-6.52) | Directly | 8 |
| Despierre  2015 | Belgium | Unclassified | I-IV | 106 | FISH | 59(31-85)  (median) | 53/106 | NA | OS | 0.97  (0.49-1.89) | Directly | 8 |
| Despierre  2015 | Belgium | Unclassified | I-IV | 106 | FISH | 59(31-85)  (median) | 53/106 | NA | PFS | 1.51  (0.87-2.63) | Directly | 8 |
| Corkery  2015 [14] | Canada | Serous | NA | 103 | IHC | NA | NA | NA | OS | 4.41  (1.95-9.95) | Indirectly | 6 |
| Corkery  2015 [14] | Canada | Serous | NA | 103 | IHC | NA | NA | NA | DFS | 1.54  (0.91-2.6) | Indirectly | 6 |
| Demir  2014 [16] | Sweden | Unclassified | I-IV | 82 | IHC | 54(24-80)  (median) | 15/82 | NA | OS | 4.9  (2-12.04) | Indirectly | 7 |
| Cai  2015 [6] | China | Unclassified | I-IV | 95 | IHC | NA | 32/95 | NA | OS | 1.34  (0.77-2.32) | Indirectly | 8 |
| Matsuo  2014 [15] | USA | Serous | I-IV | 120 | IHC | 62.6±10.6  (mean) | 32/120 | NA | OS | 1.19  (0.67-2.11) | Directly | 6 |
| Matsuo  2014 [15] | USA | Serous | I-IV | 120 | IHC | 62.6±10.6  (mean) | 32/120 | NA | PFS | 1.04  (0.63-1.71) | Directly | 6 |
| De Toledo  2013 [17] | Brazil | Unclassified | I-IV | 152 | IHC | 55.2±12.3  (mean) | 19/152 | 43.6  (mean) | OS | 1.46  (0.42-5.04) | Directly | 7 |
| De Toledo  2013 [17] | Brazil | Unclassified | I-IV | 152 | IHC | 55.2±12.3  (mean) | 19/152 | 43.6  (mean) | DFS | 1.57  (0.39-6.22) | Directly | 7 |
| Chay  2013 [18] | Singapore | Serous | I-IV | 113 | IHC | 48.3(15.8-89) (median) | 31/113 | 2.8(0-19.99)  (median) | OS | 0.56  (0.21-1.52) | Directly | 8 |
| Chay  2013 [18] | Singapore | Serous | I-IV | 113 | IHC | 48.3(15.8-89) (median) | 31/113 | 2.8(0-19.99)  (median) | PFS | 0.5  (0.2-1.22) | Directly | 8 |
| Steffensen 2011 [19] | Denmark | Unclassified | I-IV | 139 | Elisa | 64(32-84)  (median) | NA | 39.6  (median) | OS | 1.11  (0.68-1.79) | Indirectly | 7 |
| Steffensen 2011 [19] | Denmark | Unclassified | I-IV | 139 | Elisa | 64(32-84)  (median) | NA | 39.6  (median) | PFS | 1.23  (0.64-2.39) | Indirectly | 7 |
| Liu  2010 [20] | China | Unclassified | I-IV | 116 | IHC | 49(30-76)  (median) | 26/116 | 43(5-93)  (median) | OS | 2.83  (1.39-5.79) | Indirectly | 9 |
| Liu  2010 [20] | China | Unclassified | I-IV | 116 | IHC | 49(30-76)  (median) | 26/116 | 43(5-93)  (median) | PFS | 1.92  (1-3.69) | Indirectly | 9 |
| Pfisterer  2009 [21] | Germany | Unclassified | IIB-IV | 359 | IHC | ≥18 | 22/359 | 57.5(46-64.3)  (median) | OS | 0.71  (0.42-1.18) | Directly | 8 |
| Garcia-Velasco 2008 [23] | Spain | Unclassified | NA | 72 | IHC | 57(28-82)  (median) | 4/72 | 33  (median) | OS | 2.28  (0.12-4.2) | Directly | 7 |
| Garcia-Velasco 2009 [23] | Spain | Unclassified | NA | 72 | IHC | 57(28-82)  (median) | 4/72 | 33  (median) | PFS | 2.82  (0.38-20.9) | Directly | 7 |
| Graeff  2008 [24] | Netherland | Unclassified | I-IV | 230 | immunostaining | 57.8(22-90)  (median) | 12/230 | NA | OS | 1.02  (0.48-2.2) | Directly | 6 |
| Graeff  2008 [24] | Netherland | Unclassified | I-IV | 230 | immunostaining | 57.8(22-90)  (median) | 12/230 | NA | PFS | 0.98  (0.46-2.1) | Directly | 6 |
| Tomsova  2008 | Czech Republic | Unclassified | I-IV | 116 | IHC | 53(27-82)  (median) | 10/116 | 39(1-120)  (median) | OS | 1.9  (0.79-4.58) | Indirectly | 7 |
| Tuefferd  2007 [25] | France | Unclassified | I-IV | 320 | IHC | 58(25-77)  median | 41/320 | 24.9  (median) | OS | 0.95  (0.51-1.74) | Directly | 7 |
| Tuefferd  2007 [25] | France | Unclassified | I-IV | 320 | IHC | 58(25-77)  median | 41/320 | 24.9  (median) | PFS | 0.81  (0.54-1.19) | Directly | 7 |
| Steffensen  2007 | Denmark | Unclassified | II-IV | 160 | IHC | 54.5(29-70)  median | 57/160 | 120  (all) | OS | 1.5  (1.02-2.2) | Directly | 8 |
| Pils  2007 [27] | Austria | Unclassified | I-IV | 128 | IHC | 59.2  (mean) | 35/128 | 43.7(0.4-168.7)  (median) | OS | 1.92  (0.94-3.94) | Indirectly | 7 |
| Malamou-Mitsi 2007[28] | Greece | Unclassified | I-III | 95 | IHC | NA | 17/95 | 66(0.4-89.3)  (median) | OS | 1.85  (0.93-4.12) | Indirectly | 7 |
| Malamou-Mitsi 2007[28] | Greece | Unclassified | I-III | 95 | IHC | NA | 17/95 | 66(0.4-89.3)  (median) | PFS | 1.44  (0.79-2.63) | Indirectly | 7 |
| Brozek  2006 [31] | Gdansk | Unclassified | I-IV | 53 | FISH | NA | 10/53 | NA | OS | 2.44  (0.79-7.52) | Indirectly | 6 |
| Surowiak 2006 [29] | Germany | Unclassified | I-III | 43 | IHC | 51  (mean) | 21/43 | 0-52 | OS | 0.85  (0.17-4.33) | Indirectly | 7 |
| Castellvi  2006 [30] | Spain | Unclassified | I-IV | 75 | IHC | 55(20-87)  (mean) | 23/75 | 31(24-80)  (mean) | OS | 1.12  (0.49-2.54) | Indirectly | 7 |
| Verri  2005 [32] | Italy | Unclassified | I-IV | 194 | IHC | 57(25-90)  median | 53/194 | 45(1-161)  (median) | OS | 1.36  (0.76-2.42) | Directly | 8 |
| Verri  2005 [32] | Italy | Unclassified | I-IV | 194 | IHC | 57(25-90)  median | 53/194 | 45(1-161)  (median) | PFS | 1.61  (0.94-2.73) | Directly | 8 |
| Lassus  2004 [34] | Sweden | Serous | I-IV | 401 | CISH | NA | 66/401 | NA | OS | 2.14  (1.34-3.42) | Directly | 7 |
| Nielsen  2003 [33] | Denmark | Unclassified | I-IV | 783 | IHC | 58(13-91)  (median) | 272/783 | NA | OS | 0.95  (0.66-1.36) | Directly | 7 |
| Camilleri-Broet 2004 [35] | France | Unclassified | IIIA-IV | 95 | IHC | 59(23-74)  median | 15/95 | 68  (median) | OS | 2.12  (1.13-3.98) | Directly | 7 |
| Camilleri-Broet 2004 [35] | France | Unclassified | IIIA-IV | 95 | IHC | 59(23-74)  median | 15/95 | 68  (median) | PFS | 1.99  (1.12-3.54) | Directly | 7 |
| Skirnisdottir  2001[36] | Sweden | Unclassified | IA-IIC | 106 | IHC | 60(26-82)  mean | 20/106 | 87(57-125)  mean | OS | 2.28  (0.67-7.82) | Indirectly | 8 |
| Davidson  2000 [37] | Norway | Unclassified | NA | 75 | IHC | 56.9(30-84)  mean | 35/75 | 70(8-224)  mean | OS | 1.92  (1.1-3.37) | Indirectly | 8 |
| Wang  1999 [38] | USA | Unclassified | NA | 40 | FISH | 61(35-83)  median | 10/40 | 1-56  all | OS | 4  (1.2-13.9) | Indirectly | 8 |
| Medl  1995 [39] | Austria | Unclassified | I-IV | 196 | PCR | 59.6(15-88)  median | 79/196 | 59  mean | OS | 1.08  (0.73-1.6) | Indirectly | 8 |
| Fajac  1995[40] | France | Unclassified | I-IV | 65 | South blot | 52  mean | 9/65 | 71  (10-43)  median | OS | 1.8  (0.75-4.33) | Indirectly | 7 |
| Berchuck  1990 [41] | USA | Unclassified | NA | 73 | IHC | 63.5  (median) | 23/73 | 1-100  (all) | OS | 4.39  (2.13-9.06) | Inirectly | 8 |

Abbreviations: HR: hazard ratio, CI: confidence interval, IHC: immunohistochemistry, FISH: fluorescence in situ hybridization. ELISA: enzyme-linked immunosorbent assay, CISH: chromogenic in situ hybridization, PCR: polymerase chain reaction, NA: not available, OS: overall survival, DFS/PFS: disease-free survival/ progress-free survival, Serous: serous ovarian cancer, Unclassified: serous, mucinous, clear cell, endometrioid, transitional cell, undifferentiated, differentiated, and others.
